# Event-Related Brain Potentials N140 and P300 during Somatosensory Go/NoGo Tasks Are Modulated by Movement Preparation

**DOI:** 10.3390/brainsci14010038

**Published:** 2023-12-30

**Authors:** Yuya Matsuda, Yasushi Sugawara, Mayu Akaiwa, Hidekazu Saito, Eriko Shibata, Takeshi Sasaki, Kazuhiro Sugawara

**Affiliations:** 1 Graduate School of Health Sciences, Sapporo Medical University, Sapporo 060-8556, Hokkaido, Japan; 2 Department of Occupational Therapy, School of Health Science, Sapporo Medical University, Sapporo 060-8556, Hokkaido, Japan; 3 Major of Physical Therapy, Department of Rehabilitation, Faculty of Healthcare and Science, Hokkaido Bunkyo University, Eniwa 061-1449, Hokkaido, Japan; 4 Department of Physical Therapy, School of Health Science, Sapporo Medical University, Sapporo 060-8556, Hokkaido, Japan

**Keywords:** somatosensory evoked potentials, movement-related cortical potentials, N140, P300, Go/NoGo task

## Abstract

The Go/NoGo task requires attention and sensory processing to distinguish a motor action cue or ‘Go stimulus’ from a ‘NoGo stimulus’ requiring no action, as well as motor preparation for a rapid Go stimulus response. The neural activity mediating these response phases can be examined non-invasively by measuring specific event-related brain potentials (ERPs) using electroencephalography. However, it is critical to determine how different task conditions, such as the relationship between attention site and movement site, influence ERPs and task performance. In this study, we compared attention-associated ERP components N140 and P300, the performance metrics reaction time (RT) and accuracy (%Error) and movement-related cortical potentials (MRCPs) between Go/NoGo task trials in which attention target and movement site were the same (right index finger movement in response to right index finger stimulation) or different (right index finger movement in response to fifth finger stimulation). In other Count trials, participants kept a running count of target stimuli presented but did not initiate a motor response. The N140 amplitudes at electrode site Cz were significantly larger in Movement trials than in Count trials regardless of the stimulation site–movement site condition. In contrast, the P300 amplitude at Cz was significantly smaller in Movement trials than in Count trials. The temporal windows of N140 and P300 overlapped with the MRCP. This superposition may influence N140 and P300 through summation, possibly independent of changes in attentional allocation.

## 1. Introduction

Motor reaction tasks requiring movement in response to external sensory stimuli (such as visual, auditory or somatosensory stimuli) are widely used to evaluate human sensorimotor and attentional functions [1,2,3,4]. In the typical sensorimotor version of the Go/NoGo task, subjects respond with a key press to a specific ‘Go’ stimulus in randomly ordered Go trials but must refrain from responding to an alternative ‘NoGo’ stimulus in the remaining NoGo trials. Accurate and rapid Go/NoGo task performance requires attention to the stimulus and motor preparation in order to respond as quickly as possible. Moreover, coupling these Go/NoGo tasks with modern neuroimaging modalities or non-invasive electrophysiological methods such as electroencephalographic (EEG) measurements can reveal important aspects of cortical sensorimotor processing and allocation of attention [3,5,6].

The EEG recorded during Go/NoGo and other sensorimotor tasks is characterised by a series of waveforms reflecting individual neural processing steps, including event-related brain potentials (ERPs) evoked by external stimuli [7]. The somatosensory N140 is a negative waveform appearing on central EEG electrodes about 100–180 ms after skin stimulation and is functionally analogous to the auditory or visual N1 ERP reflecting sensory gating by attention [8,9,10,11]. The ensuing P300 is a positive waveform peaking 300–500 ms after external stimulation that reflects the allocation of attentional resources to external stimuli regardless of sensory modality [12,13,14,15,16,17,18,19,20] as well as working memory and context updating [21,22]. In tasks requiring motor responses, movement-related cortical potentials (MRCPs) also appear over the primary motor cortex and supplementary motor area ~1–2 s prior to movement onset [23,24,25,26]. There is a negative correlation between the MRCP amplitude and reaction time from external stimulus to movement onset, indicating a consistent relationship between movement preparation and movement performance [27]. However, the relationship between ERP component characteristics and movement execution remains a matter of debate.

Somatosensory information is especially critical for actions requiring hand dexterity [28], such as using tools (e.g., chopsticks) or operating a keyboard, and successful execution requires that attentional resources be allocated precisely to sensory processing and motor performance during different phases of the activity. For instance, attention allocation has been shown to increase input to the somatosensory cortex and improve hand motor performance [29]. Previous studies have also reported that the P300 amplitude is smaller during a stimulus-cued movement task than a stimulus counting task [4,15], suggesting that attentional allocation to the stimulus is reduced in order to allocate additional attention resources to the movement. However, the expected changes in N140 or P300 are unclear because the stimulation and movement sites were always different across these studies (i.e., the relationship between sites was constant). It is possible that the participants may have increased attentional allocation to the movement site and reduced attentional allocation to the stimulation site to achieve high motor performance. Indeed, it has been shown that attentional modulation is affected by the distance between the stimulation site and site of attention [30] and that cortical responses are greater when stimuli are presented to the attentive finger compared to the non-attentive finger [31]. Alternatively, waveforms may be altered by simple summation.

Therefore, in this study, we investigated the influence of congruence between somatosensory stimulation site and movement site on ERPs during a Go/NoGo task with two trial conditions, a Congruent condition where the sensory stimulation site and movement site are the same (both right index finger) and an Incongruent condition when stimulation site and movement site are different (fifth finger stimulation and index finger movement). We hypothesised that in the Congruent condition, attention directed to the movement would be enhanced and ERP amplitudes reflecting attention to the stimulus would increase. Furthermore, as the above enhancements are due to the movement, the Count task (without movement) makes no difference between the two conditions.

## 2. Materials and Methods

### 2.1. Participants

The minimum sample size for the present study was determined using G*power software (version 3.1) from partial η-squared as was determined in a previous study [31]. The effect size was fixed at 0.35. This analysis revealed a minimum sample size of 17. Accordingly, 17 healthy young adults (age [mean ± standard deviation (SD)], 22.2 ± 1.3 years; all right-handed; 11 males, 6 females) participated in this study, and all participants provided written informed consent. None of the participants engaged in recreational drug use or used medication that affected the central nervous system.

### 2.2. Experimental Procedures

**Figure 1:**
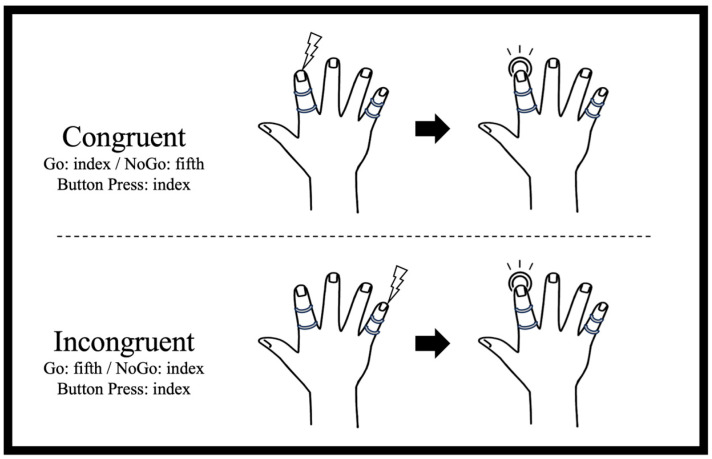
Trial conditions.

Subjects completed two Go/NoGo tasks, Count and Movement, and the Movement task was conducted with two trial conditions, Congruent (same stimulation and movement site) and Incongruent (different stimulation and movement sites) (Figure 1). All participants rested their arms comfortably on the armrests of a plastic table with the elbow joints flexed 45–50 degrees, hands in full pronation, and all fingers and thumb extended naturally. During the recordings, the subjects were instructed to keep their eyes open and look at a fixation point located in front of them at approximately 1 m. In the Movement trials (Congruent and Incongruent), the participants pressed a button with their right index finger as quickly as possible only after presentation of the target (Go) stimulus. In the Count task, the participants counted the number of target (Go) stimuli presented and were given feedback at the end of each trial block to encourage a high level of accuracy. Thus, the participants simply directed their attention to the target stimuli without any movement, such as focusing on the stimuli presented to index finger in the Congruent condition and on the stimuli presented to little finger in the Incongruent condition. Stimulus probabilities were set at 20% Go and 80% NoGo in each task block. In Count task blocks, the number of target (Go) stimuli was varied slightly (from 0 to 4 more or less) to prevent participants from predicting the number without counting.

One block consisted of 200 trials, and participants completed three blocks each of Count and Movement task trials. The inter-stimulus interval was set at 2 s in all blocks, and the inter-block interval was determined by the participant. We measured ERPs during all trials as well as MRCPs and RTs during Movement task trials. Block order was pseudorandom on the same day. All participants performed several practice trials prior to the recordings with the target stimulus at 50% probability in Movement trial blocks. The duration of the experiment was approximately 2 h, excluding the recording preparation.

### 2.3. Stimulation

Participants wore ring electrodes on the index and fifth fingers of the right hand for somatosensory stimulation. The stimulus was constant-current square-wave pulse 0.2-ms in duration, and intensity was set to three times the sensory threshold [11], which produced no pain or unpleasant sensation. The anode was placed at the distal interphalangeal joint and the cathode at the proximal interphalangeal joint of the corresponding finger. In the congruent condition, somatosensory stimulus was presented to the index finger as a Go stimulus and to the fifth finger as a NoGo stimulus. However, in the Incongruent condition, stimulus was presented to the fifth finger as a Go stimulus and to the index finger as a NoGo stimulus.

### 2.4. Electroencephalography (EEG) Recordings

All electrophysiological recordings were acquired using the Neuropack X1 MEB-2312 (Nihon Kohden, Tokyo, Japan). Electroencephalograms were recorded with Ag/AgCl disk electrodes placed on the scalp at Fz, C3, Cz, C4 and Pz positions according to the International 10–20 System. Each scalp electrode was referenced to linked earlobes (A1A2) and impedance was maintained at less than 5 kΩ. Data were band-pass filtered at 0.1–200 Hz and digitised at 1000 Hz. The electrooculogram (EOG) was recorded from the right suborbital region to eliminate eye movements or blinks exceeding 100 mV [15]. The stimulus onset was set at 0 ms, and the analysis epoch for ERPs following stimulus was set at 750 ms, including a pre-stimulus baseline period of 100 ms. The N140 and P300 peaks were analysed within time windows of 100–180 ms [11,32] and 250–650 ms [17], respectively. Amplitudes were measured by taking the lowest and highest values within the corresponding time windows and then subtracting the pre-stimulus baseline. The latencies were measured from stimulus onset to peaks.

In addition to ERPs, MRCPs were recorded to investigate the relationships between motor preparation and ERPs reflecting attentional function. In Movement task trials (both Congruent and Incongruent), we obtained MRCP waveforms off-line using the onset of the response (button pressing) as a trigger. The MRCP analysis period was from 2000 ms before to 500 ms after movement onset, with the first 400 ms (from −2000 to −1600 ms before movement onset) used to calculate baseline MRCP amplitude. Then, to investigate the effects of ERPs on MRCPs, the MRCP amplitude at the times of peaks N140 and P300 was calculated for each participant.

All participants completed more than 100 ERP and 80 MRCP measurement trials. However, the EEG data of two participants were excluded from the analysis due to numerous artefacts from blinks and unintended muscle contractions. Therefore, we analysed the ERPs and MRCP data from 15 participants.

### 2.5. Behavioural Data Recordings

The RT was defined as the time from Go stimulus onset to button pressing onset (in ms). Slow responses with RT exceeding 600 ms [11] and incorrect responses were eliminated from the averaging process. Additionally, the error rates in Movement task blocks were calculated as a measure of response accuracy.

### 2.6. Statistical Analysis

All analyses were performed using SPSS (version 24). All data were first assessed for normality using the Shapiro–Wilk test (*p* < 0.001) and subsequently expressed as mean ± SD. Differences in peak ERP amplitude and latency were evaluated by two-way analysis of variance (ANOVA) with main factors Movement condition (Congruent vs. Incongruent) and task (Movement vs. Count). Error rate, RT and MRCP metrics were compared by paired sample *t*-test. *p* < 0.05 was considered significant for all tests.

## 3. Results

### 3.1. Effects of Go/NoGo Task Condition on Performance

**Figure 2:**
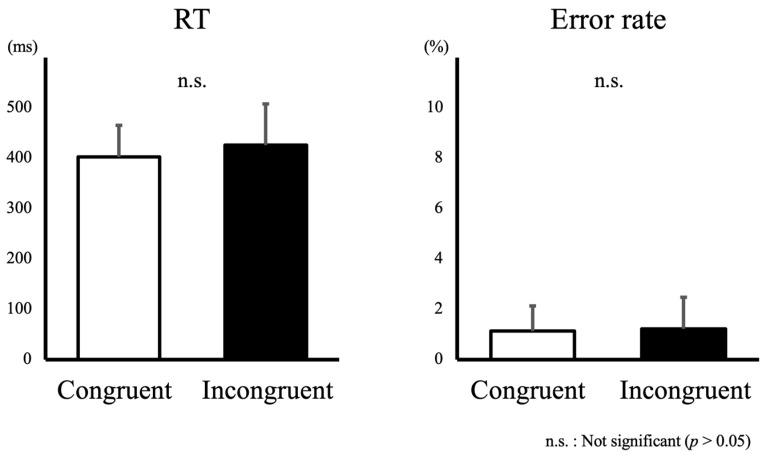
Mean reaction times (RTs) and error rates in each task and Movement task condition.

The mean RT was slightly shorter in the Congruent condition than in the Incongruent condition, but the difference did not reach statistical significance (402 ± 62 vs. 426 ± 81 ms; t (14) = −0.696, *p* = 0.112). Similarly, the error rate was approximately the same under Congruent and Incongruent conditions (1.1% ± 1.0% vs. 1.2% ± 1.3%; t (14) = −0.462, *p* = 0.651) (Figure 2).

### 3.2. Effects of Task and Task Condition on ERP Waveforms

**Figure 3:**
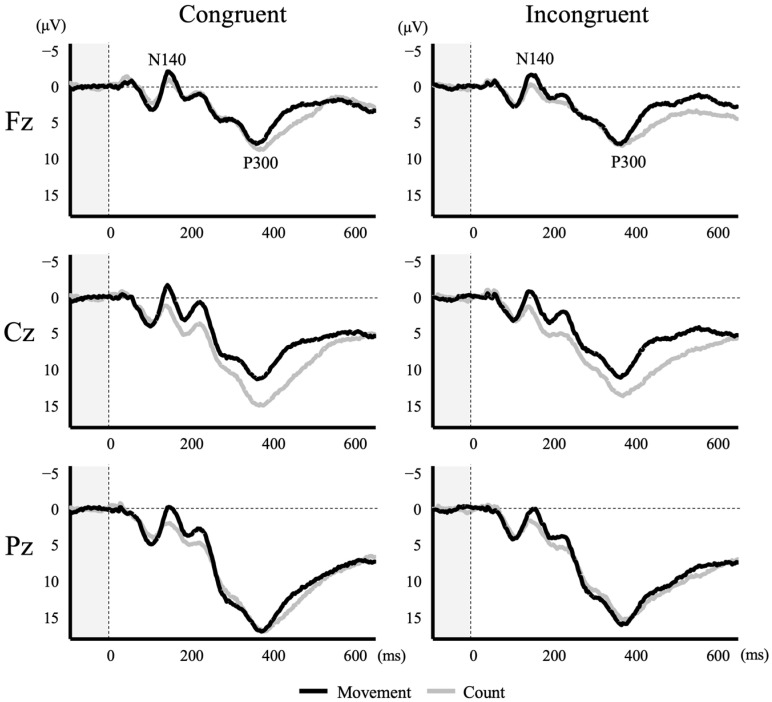
Grand average waveforms of ERPs following somatosensory stimulation at Fz, Cz and Pz triggered by target (Go) stimulus.

The average number of epochs accepted for the ERP analysis was greater than 100 in all trial types (Congruent: 106 ± 5 in Movement trials, 107 ± 6 in the Count trials; Incongruent condition: 107 ± 5 in Movement trials, 107 ± 6 in Count trials). Figure 3 presents the grand average ERP waveforms at Fz, Cz and Pz electrode sites triggered by target (Go) stimulus. Both N140 and P300 were evoked in all trial types. Peak amplitudes and latencies at Fz, Cz and Pz are shown in Table 1 and Table 2, respectively.

### 3.3. Differences in N140 Amplitude and Latency between Tasks and Conditions

A two-way ANOVA revealed a significant main effect of task (Movement vs. Count) on amplitude at Cz (F (1, 56) = 5.290, *p* = 0.025), with a greater amplitude during the Movement task than the Count task (Table 1). On the other hand, there was no significant main effect of Movement task condition (Congruent vs. Incongruent) and no task × condition interaction at any electrode site (*p* > 0.05). Also, a two-way ANOVA revealed no significant main effect of task or condition on N140 latency and no significant task × condition interaction at any electrode site (*p* > 0.05) (Table 2).

### 3.4. Differences in P300 Amplitude and Latency between Tasks and Conditions

Task and condition had similar effects on P300. A two-way ANOVA revealed a significant main effect of task on P300 amplitude at Cz (F (1, 56) = 4.191, *p* = 0.045), with significantly reduced amplitude in the Movement task than the Count task (Table 1). On the other hand, there was no significant main effect of condition and no significant task × condition interaction effect on amplitude at any electrode site (*p* > 0.05). Also, two-way ANOVA revealed no significant main effect of task or condition on P300 latency and no significant task × condition interaction effect at any electrode site (*p* > 0.05) (Table 2).

### 3.5. Effects of MRCPs on N140 and P300

**Figure 4:**
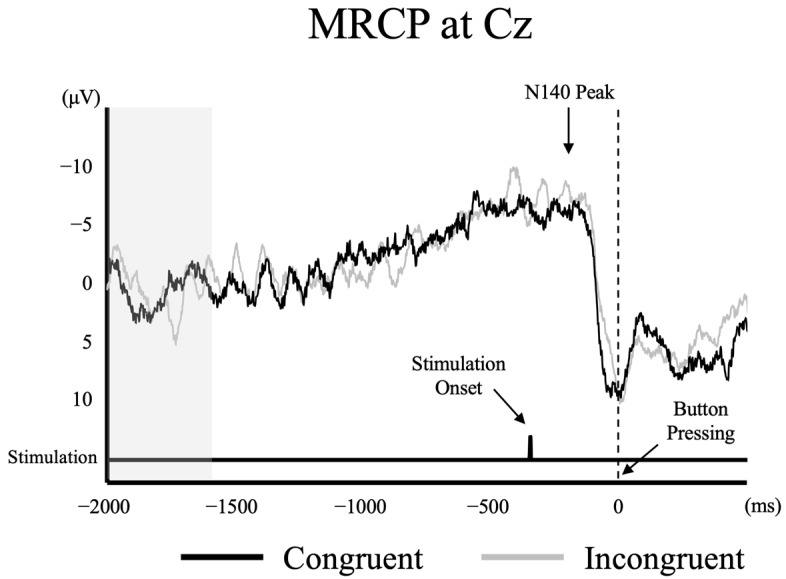
The grand-averaged MRCP waveform obtained from Cz in a representative participant.

The average number of epochs accepted for MRCP analysis was greater than 80 for both conditions (Congruent condition: 92.7 ± 11.4; Incongruent condition: 93.6 ± 12.6). Figure 4 shows the grand average MRCP waveforms obtained from the Cz of a representative participant in both Congruent and Incongruent conditions and the average time of N140 peak from all trials. The time of N140 peak coincided with the period of increasing (more negative) MRCP amplitude, and MRCP amplitude at peak N140 and P300 did not differ significantly between Congruent and Incongruent conditions (At peak N140: −2.4 ± 5.3 eve vs. −3.3 ± 4.9 eve; (t (14) = −0.975, *p* = 0.346; At peak P300: 6.0 ± 6.2 eve vs. 4.2 ± 5.0 eve; (t (14) = 1.563, *p* = 0.140).

## 4. Discussion

This is the first study to examine the effects of stimulation site–movement site congruence in a somatosensory Go/NoGo task on ERPs reflecting attentional function (N140 and P300). The present results revealed that behavioural performance did not differ significantly between Congruent (same site) and Incongruent (different site) conditions. ERP amplitudes did not differ significantly between the two conditions; however, the N140 amplitude at Cz was significantly greater, and the P300 amplitude at Cz was significantly smaller under both the conditions compared to a non-movement Count task. Further, we demonstrated that these ERPs overlapped temporally with MRCPs during movement preparation in the movement task. Thus, the attentional allocation may not be enhanced due to congruence between the movement and stimulation sites. Furthermore, cortical motor preparation activity may influence ERPs through passive summation or active neural network interactions, potentially independent of attentional allocation.

It has been reported that a somatosensory-evoked N140 amplitude is enhanced when attention is directed towards the stimulus [8,9,11,33,34] and is modulated by stimulus intensity, task difficulty and stimulus interval as well as spatial attention [10,33,35,36,37]. From these findings, it has been demonstrated that N140 is not only influenced by endogenous factors such as selective attention but also affected by exogenous factors, i.e., inputs from the somatosensory system. The present results revealed a significant N140 amplitude increase at Cz in the Movement task compared to a non-movement (Count) task regardless of task condition (congruent or incongruent), consistent with a previous study in which the stimulation and movement sites were incongruent [11]. In the Go/NoGo task used in this study, it was necessary to selectively allocate attention to the Go stimulus while ignoring the NoGo stimulus regardless of whether the task was a Movement or Count task. Our finding that the N140 amplitude was increased in the movement task with movement after the presentation of the Go stimulus suggests that neural activity associated with motor execution may have influenced the attention directed to the stimulus. Further, the N140 following somatosensory stimulation is generated mainly by the anterior cingulate cortex (ACC) [38,39]. The reaction time in this study was approximately 410 ms, and the N140 peak latency overlapped with the motor preparation phase for button pressing (the required motor response), which reflects activity in the supplementary motor cortex and cingulate gyrus [40,41,42,43,44]. These results suggest that neural activity occurring during movement preparation may increase the N140 amplitude during the Movement task via a summating negative shift. As another interpretation, considering N140 has also been reported to reflect the cortical activation of the posterior parietal area [45], and the fact that N140, which may be affected by exogenous factors, was modulated during the latency of motor readiness potential generation in the present study suggests that exercise may affect somatosensory processing.

The P300 amplitude increases in parallel with the attentional resources allocated for tasks and stimuli [12,13,14,15,16,17,18,19,20]. In addition to attention, however, P300 amplitude is modulated by working memory and context updating [21,22]. In the present study, the P300 amplitude at Cz was reduced in the Movement task compared to the Count task, as in a previous study [15]. The amplitude reduction of the P300 should be interpreted with caution, as it may not simply indicate a reduction in attentional allocation to the stimulus. One possible interpretation of this result is that it reflects a difference in the working memory loads. In the Count task, the subject was required to continually update the number of congruent (target) stimuli in working memory, whereas there was no such working memory load in the Movement task [15]. It is possible that this difference in task characteristics contributed to the P300 amplitude reduction. Another interpretation is that attentional resources may have been allocated to the movement. In the present study, however, the dual task of allocating attention to movement and stimulation sites was distinguished by including congruent and incongruent conditions. Some studies have reported that the P300 amplitude is proportional to the attentional resources devoted to a given task [46,47] and decreases in more difficult tasks, such as dual tasks compared to single tasks [48]. It has also been reported that P300 evoked by somatosensory stimulation is attenuated in motor tasks that require motor control and muscle output compared to regular periodic exercise, indicating that the distribution of attention is altered by the motor task [19]. In the present study, we used a response task with stimuli to different fingers in the same hand, which may have enhanced attention to the movement rather than to the external stimuli because attention was distributed to the movement itself. Finally, summation may also account for the reduction in (positive) P300 amplitude. Additional studies are needed to distinguish among these possibilities.

The time of peak N140 coincided with the period of increasing MRCP amplitude, and the period during which P300 appeared, likewise, coincided with the period of increasing MRCP amplitude, although the time of peak did not completely coincide. Ultimately, the time windows of N140 and P300 overlapped with the period of increasing motor readiness as manifested by the slow negative potential shift. Although several previous studies have suggested that the ERP modulation is due to the superimposed MRCP [4,11], no studies have measured ERP and MRCP simultaneously. Many studies [23,25,49,50,51,52,53,54] have reported that MRCP (e.g., Bereitschaftspotential, readiness potential) is mainly observed at the Cz electrode. Then, we observed that the negative N140 amplitude increased while the positive P300 amplitude decreased at Cz electrode in the Movement task regardless of condition. Thus, these findings support previous studies reporting that the effects on ERPs are due to the superposition of the MRCP [15,55].

The limitations of this study include a focus on the relationship between attention and movement within one hand. Therefore, further studies are needed to examine the relationship between attention and movement in a wider range of sites (e.g., left and right hands, limbs, etc.). In addition, we did not perform a task in which all stimuli were ignored due to the potential for participant fatigue. The present study investigated the effect of movement on attention using the state of attention as the criterion because ERP modulation by the presence or absence of conscious attention has been investigated in many previous studies. Furthermore, the amplitude of P300 decreases with age [56]. It has also been shown that cognitive functions such as working memory and attentional control (inhibition of unnecessary stimuli) decline with ageing [57,58]. In the present study, we used young adults without attentional dysfunction, which may have allowed us to appropriately allocate attention to the stimulus site without being affected by the congruence or incongruence with the motor site. We believe that examining whether the results of this study can be generalised to older adults in the future is necessary. Last, in the present study, only about 1% of the thoughts did not respond to Go; therefore, it was difficult to directly address the point that no movement occurred in response to Go stimuli. However, to find a solution to this point, we need to investigate how ERPs change when the ratio of Go to NoGo stimuli is varied and the motor readiness potential is changed.

## 5. Conclusions

The somatosensory N140 and P300 were not affected by incongruence between the movement site and stimulation site but were altered by the waveform associated with movement preparation, possibly through passive summation. When humans execute movements in response to somatosensory stimuli, they do not necessarily reallocate attention to the movement site, but movement intention may automatically shift attention away from sensory stimuli.

## Figures and Tables

**Table 1:** Average ERP amplitudes at Fz, Cz and Pz.

Amplitude (µV)	Congruent		Incongruent	
	Movement	Count	Movement	Count
N140				
Fz	−2.8 (3.5)	−1.6 (2.7)	−2.9 (2.5)	−1.2 (1.7)
Cz	−2.1 (5.2) *	0.3 (3.5)	−1.7 (4.1) *	0.7 (2.6)
Pz	−0.6 (4.2)	1.0 (2.7)	−0.5 (3.3)	1.0 (2.9)
P300				
Fz	9.3 (5.3)	10.0 (3.8)	9.3 (3.6)	9.8 (3.7)
Cz	13.9 (5.1) *	17.0 (5.0)	13.1 (4.7) *	15.8 (6.1)
Pz	19.2 (6.4)	18.5 (5.7)	17.8 (6.6)	17.0 (7.0)

Data are expressed as mean (standard deviation). * *p* < 0.05: Movement vs. Count.

**Table 2:** Average ERP latencies at Fz, Cz and Pz.

Latency (ms)	Congruent		Incongruent	
	Movement	Count	Movement	Count
N140				
Fz	145.4 (10.9)	142.9 (6.7)	143.5 (10.7)	147.8 (16.7)
Cz	145.5 (17.2)	144.1 (19.0)	145.4 (20.0)	148.0 (26.9)
Pz	147.5 (13.3)	144.1 (13.7)	149.2 (13.9)	152.6 (24.5)
P300				
Fz	338.2 (40.4)	345.0 (44.3)	341.9 (32.2)	345.1 (31.5)
Cz	343.5 (41.8)	346.9 (42.6)	343.9 (32.4)	347.1 (33.2)
Pz	349.6 (38.5)	356.0 (39.2)	353.5 (31.6)	347.6 (35.4)

Data are expressed as mean (standard deviation).

## Data Availability

The data presented in this study are available on request from the corresponding author. The data are not publicly available due to privacy reasons.

## References

[1] Kamitani T., Kuroiwa Y., Li M., Ikegami T., Matsubara S. (2003). Relationship between cerebellar size and variation of reaction time during a visual cognitive task in normal subjects. J. Neurol..

[2] Barutchu A., Spence C. (2021). Top-down task-specific determinants of multisensory motor reaction time enhancements and sensory switch costs. Exp. Brain Res..

[3] Sugawara K., Onishi H., Yamashiro K., Soma T., Oyama M., Kirimoto H., Tamaki H., Murakami H., Kameyama S. (2013). Repeated practice of a Go/NoGo visuomotor task induces neuroplastic change in the human posterior parietal cortex: An MEG study. Exp. Brain Res..

[4] Barrett G., Neshige R., Shibasaki H. (1987). Human auditory and somatosensory event-related potentials: Effects of response condition and age. Electroencephalogr. Clin. Neurophysiol..

[5] Kida T., Wasaka T., Nakata H., Akatsuka K., Kakigi R. (2006). Active attention modulates passive attention-related neural responses to sudden somatosensory input against a silent background. Exp. Brain Res..

[6] Nakata H., Inui K., Wasaka T., Tamura Y., Akatsuka K., Kida T., Kakigi R. (2006). Higher anticipated force required a stronger inhibitory process in go/nogo tasks. Clin. Neurophysiol..

[7] Brázdil M., Roman R., Daniel P., Rektor I. (2003). Intracerebral somatosensory event-related potentials: Effect of response type (button pressing versus mental counting) on P3-like potentials within the human brain. Clin. Neurophysiol..

[8] Desmedt J.E., Robertson D. (1977). Differential enhancement of early and late components of the cerebral somatosensory evoked potentials during forced-paced cognitive tasks in man. J. Physiol..

[9] García-Larrea L., Lukaszewicz A.C., Mauguière F. (1995). Somatosensory responses during selective spatial attention: The N120-to-N140 transition. Psychophysiology.

[10] Nakajima Y., Imamura N. (2000). Relationships between attention effects and intensity effects on the cognitive N140 and P300 components of somatosensory ERPs. Clin. Neurophysiol..

[11] Kida T., Nishihira Y., Wasaka T., Nakata H., Sakamoto M. (2004). Passive enhancement of the somatosensory P100 and N140 in an active attention task using deviant alone condition. Clin. Neurophysiol..

[12] Duncan-Johnson C.C., Donchin E. (1977). On quantifying surprise: The variation of event-related potentials with subjective probability. Psychophysiology.

[13] Wickens C., Kramer A., Vanasse L., Donchin E. (1983). Performance of concurrent tasks: A psychophysiological analysis of the reciprocity of information-processing resources. Science.

[14] Polich J., Bondurant T. (1997). P300 sequence effects, probability, and interstimulus interval. Physiol. Behav..

[15] Kida T., Nishihira Y., Hatta A., Wasaka T. (2003). Somatosensory N250 and P300 during discrimination tasks. Int. J. Psychophysiol..

[16] Polich J. (2007). Updating P300: An integrative theory of P3a and P3b. Clin. Neurophysiol..

[17] Verleger R., Grauhan N., Śmigasiewicz K. (2016). Go and no-go P3 with rare and frequent stimuli in oddball tasks: A study comparing key-pressing with counting. Int. J. Psychophysiol..

[18] Akaiwa M., Iwata K., Saito H., Sasaki T., Sugawara K. (2020). Altered somatosensory evoked potentials associated with improved reaction time in a simple sensorimotor response task following repetitive practice. Brain Behav..

[19] Akaiwa M., Iwata K., Saito H., Shibata E., Sasaki T., Sugawara K. (2022). The effect of pedaling at different cadence on attentional resources. Front. Hum. Neurosci..

[20] Kida T., Nishihira Y., Hatta A., Wasaka T., Nakata H., Sakamoto M., Nakajima T. (2003). Changes in the somatosensory N250 and P300 by the variation of reaction time. Eur. J. Appl. Physiol..

[21] Donchin E., Coles M.G.H. (1988). Is the P300 component a manifestation of context updating?. Behav. Brain Sci..

[22] Linden D.E. (2005). The p300: Where in the brain is it produced and what does it tell us?. Neuroscientist.

[23] Cui R.Q., Deecke L. (1999). High resolution DC-EEG of the bereitschaftspotential preceding anatomically congruent versus spatially congruent bimanual finger movements. Brain Topogr..

[24] Di Russo F., Incoccia C., Formisano R., Sabatini U., Zoccolotti P. (2005). Abnormal motor preparation in severe traumatic brain injury with good recovery. J. Neurotrauma.

[25] Di Russo F., Lucci G., Sulpizio V., Berchicci M., Spinelli D., Pitzalis S., Galati G. (2016). Spatiotemporal brain mapping during preparation, perception, and action. Neuroimage.

[26] Sulpizio V., Lucci G., Berchicci M., Galati G., Pitzalis S., Di Russo F. (2017). Hemispheric asymmetries in the transition from action preparation to execution. Neuroimage.

[27] Haagh S.A., Brunia C.H. (1985). Anticipatory response-relevant muscle activity, CNV amplitude and simple reaction time. Electroencephalogr. Clin. Neurophysiol..

[28] Rothwell J.C., Traub M.M., Day B.L., Obeso J.A., Thomas P.K., Marsden C.D. (1982). Manual motor performance in a deafferented man. Brain.

[29] Rossettini G., Testa M., Vicentini M., Manganotti P. (2017). The effect of different attentional focus instructions during finger movement tasks in healthy subjects: An exploratory study. BioMed Res. Int..

[30] Heed T., Röder B. (2010). Common anatomical and external coding for hands and feet in tactile attention: Evidence from event-related potentials. J. Cogn. Neurosci..

[31] Kida T., Tanaka E., Kakigi R. (2018). Adaptive flexibility of the within-hand attentional gradient in touch: An MEG study. Neuroimage.

[32] Lindenbaum L., Zehe S., Anlauff J., Hermann T., Kissler J.M. (2021). Different patterns of attention modulation in early N140 and late P300 sERPs following ipsilateral vs. contralateral stimulation at the fingers and cheeks. Front. Hum. Neurosci..

[33] Michie P.T., Bearpark H.M., Crawford J.M., Glue L.C. (1987). The effects of spatial selective attention on the somatosensory event-related potential. Psychophysiology.

[34] Kekoni J., Hämäläinen H., McCloud V., Reinikainen K., Näätänen R. (1996). Is the somatosensory N250 related to deviance discrimination or conscious target detection?. Electroencephalogr. Clin. Neurophysiol..

[35] Nakajima Y., Imamura N. (2000). Probability and interstimulus interval effects on the N140 and the P300 components of somatosensory erps. Int. J. Neurosci..

[36] Eimer M., Forster B. (2003). The spatial distribution of attentional selectivity in touch: Evidence from somatosensory ERP components. Clin. Neurophysiol..

[37] Eimer M., Forster B. (2003). Modulations of early somatosensory ERP components by transient and sustained spatial attention. Exp. Brain Res..

[38] Waberski T.D., Gobbelé R., Darvas F., Schmitz S., Buchner H. (2002). Spatiotemporal imaging of electrical activity related to attention to somatosensory stimulation. Neuroimage.

[39] Tanaka E., Inui K., Kida T., Miyazaki T., Takeshima Y., Kakigi R. (2008). A transition from unimodal to multimodal activations in four sensory modalities in humans: An electrophysiological study. BMC Neurosci..

[40] Cui R.Q., Deecke L. (1999). High resolution DC-EEG analysis of the bereitschaftspotential and post movement onset potentials accompanying uni- or bilateral voluntary finger movements. Brain Topogr..

[41] Ball T., Schreiber A., Feige B., Wagner M., Lücking C.H., Kristeva-Feige R. (1999). The role of higher-order motor areas in voluntary movement as revealed by high-resolution EEG and fMRI. Neuroimage.

[42] Yazawa S., Ikeda A., Kunieda T., Mima T., Nagamine T., Ohara S., Terada K., Taki W., Kimura J., Shibasaki H. (1998). Human supplementary motor area is active in preparation for both voluntary muscle relaxation and contraction: Subdural recording of bereitschaftspotential. Neurosci. Lett..

[43] Ikeda A., Shibasaki H., Nagamine T., Terada K., Kaji R., Fukuyama H., Kimura J. (1994). Dissociation between contingent negative variation and bereitschaftspotential in a patient with cerebellar efferent lesion. Electroencephalogr. Clin. Neurophysiol..

[44] Lang W., Cheyne D., Kristeva R., Beisteiner R., Lindinger G., Deecke L. (1991). Three-dimensional localization of SMA activity preceding voluntary movement. A study of electric and magnetic fields in a patient with infarction of the right supplementary motor area. Exp. Brain Res..

[45] Tomberg C. (1999). Cognitive N140 electrogenesis and concomitant 40 Hz synchronization in mid-dorsolateral prefrontal cortex (area 46) identified in non-averaged human brain potentials. Neurosci. Lett..

[46] Sutton S., Braren M., Zubin J., John E.R. (1965). Evoked-potential correlates of stimulus uncertainty. Science.

[47] Kok A. (2001). On the utility of P3 amplitude as a measure of processing capacity. Psychophysiology.

[48] Kida T., Kaneda T., Nishihira Y. (2012). Modulation of somatosensory processing in dual tasks: An event-related brain potential study. Exp. Brain Res..

[49] Houdayer E., Lee S.J., Hallett M. (2020). Cerebral preparation of spontaneous movements: An EEG study. Clin. Neurophysiol..

[50] Kornhuber H.H., Deecke L. (2016). Brain potential changes in voluntary and passive movements in humans: Readiness potential and reafferent potentials. Pflugers. Arch..

[51] Cui R.Q., Huter D., Lang W., Deecke L. (1999). Neuroimage of voluntary movement: Topography of the bereitschaftspotential, a 64-channel DC current source density study. Neuroimage.

[52] Nagamine T., Kajola M., Salmelin R., Shibasaki H., Hari R. (1996). Movement-related slow cortical magnetic fields and changes of spontaneous MEG- and EEG-brain rhythms. Electroencephalogr. Clin. Neurophysiol..

[53] Neshige R., Lüders H., Friedman L., Shibasaki H. (1988). Recording of movement-related potentials from the human cortex. Ann. Neurol..

[54] Ikeda A., Shibasaki H., Kaji R., Terada K., Nagamine T., Honda M., Kimura J. (1997). Dissociation between contingent negative variation (CNV) and bereitschaftspotential (BP) in patients with parkinsonism. Electroencephalogr. Clin. Neurophysiol..

[55] Nakata H., Inui K., Nishihira Y., Hatta A., Sakamoto M., Kida T., Wasaka T., Kakigi R. (2004). Effects of a go/nogo task on event-related potentials following somatosensory stimulation. Clin. Neurophysiol..

[56] Walhovd K.B., Rosquist H., Fjell A.M. (2008). P300 amplitude age reductions are not caused by latency jitter. Psychophysiology.

[57] Commodari E., Guarnera M. (2008). Attention and aging. Aging Clin. Exp. Res..

[58] Verhaeghen P., Cerella J. (2002). Aging, executive control, and attention: A review of meta-analyses. Neurosci. Biobehav. Rev..

